# Car Driving Status and Living Arrangement Associated with Sarcopenia among Rural Japanese Older Adults: A Cross-Sectional Study

**DOI:** 10.3390/ijerph19010414

**Published:** 2021-12-31

**Authors:** Tsuyoshi Hamano, Takafumi Abe, Ryo Miyazaki, Kenta Okuyama, Kristina Sundquist, Toru Nabika

**Affiliations:** 1 Department of Sports Sociology and Health Sciences, Faculty of Sociology, Kyoto Sangyo University, Kyoto 603-8555, Japan; 2 Center for Community-Based Healthcare Research and Education (CoHRE), Head Office for Research and Academic Information, Shimane University, Izumo 693-8501, Japan; t-abe@med.shimane-u.ac.jp (T.A.); miyazaki@hmn.shimane-u.ac.jp (R.M.); kenta.okuyama@med.lu.se (K.O.); kristina.sundquist@med.lu.se (K.S.); nabika@med.shimane-u.ac.jp (T.N.); 3 Faculty of Human Sciences, Shimane University, Matsue 690-8504, Japan; 4 Center for Primary Health Care Research, Department of Clinical Sciences Malmö, Lund University, 20502 Malmö, Sweden; 5 Department of Family Medicine and Community Health, Department of Population Health Science and Policy, Icahn School of Medicine at Mount Sinai, New York, NY 10029-5674, USA; 6 Department of Functional Pathology, Faculty of Medicine, Shimane University, Izumo 693-8501, Japan

**Keywords:** rural area, sarcopenia, car driving status, living arrangement, older adults

## Abstract

Ensuring mobility after driving cessation is an important public health issue to prevent functional limitations, but this issue is still not fully understood in rural settings. The aim of this study was to test the hypothesis that being a non-driver and living alone is associated with a greater risk of sarcopenia among the community-dwelling elderly in rural Japanese areas. This study was conducted in 2018 and data from 738 participants were used. Sarcopenia was assessed by measuring walking speed, handgrip strength, and skeletal muscle mass. Car driving status and living arrangement were collected using self-reported questionnaires and face-to-face interviews. Four groups were set to determine combined conditions of car driving status and living arrangement. Logistic regression analysis was performed to estimate the odds ratio (OR) and a 95% confidence interval of sarcopenia after adjustment for confounding factors. Compared with the reference group (driver and living with others), the OR of sarcopenia was significantly higher in the non-driver and living alone group (OR = 2.21; 95% confidence interval, 1.02–4.80). Our findings suggest that the consideration of both driving status and living arrangement are important in the formulation of public health strategies to prevent sarcopenia in rural Japanese areas.

## 1. Introduction

Driving cessation in older adults is associated with a variety of health outcomes [1]. Previous cohort studies have revealed an association between driving cessation and an increased risk of functional limitations [2,3]. Given that driving cessation leads to a decline in out-of-home activity levels, developing a range of acceptable transportation options is necessary to maintain the independence of older adults [4]. In rural areas, car driving has become the primary means of transportation, since the public transport network is often more inconvenient than in urban areas [5]. In Japan, drivers aged 75 years or older have been required to have cognitive screenings when they renew their license since June 2009 [6]. Furthermore, older drivers are required to voluntarily return their licenses if they are unable to drive safely. Therefore, ensuring mobility after driving cessation in rural settings is an important public health issue to prevent functional limitations.

A recent study from Japanese rural areas focused on outing support for older adults. It revealed that those who were non-drivers were more likely to request a family member living with them rather than a neighbor to provide transportation to shop for daily necessities or to access medical care [7]. Given that older adults who live alone are more likely to experience problems with respect to mobility, this situation may lead to increasing functional limitations. Sarcopenia is prevalent among older people and its severity is positively and independently associated with the degree of functional limitation [8]. An early diagnosis of sarcopenia is effective for identifying vulnerable community-dwelling older adults at an early stage of the disabling process [8].

To our knowledge, no studies have examined the combined condition of car driving status (drivers or non-drivers) and possibility of assistance to obtain transportation (living with others or living alone) on sarcopenia. With this background, the aim of this study is to test the hypothesis that being a non-driver and living alone is associated with a greater risk of sarcopenia among community-dwelling older adults in Japanese rural areas.

## 2. Materials and Methods

### 2.1. Data

This cross-sectional study used data from older adults who participated in the Shimane CoHRE study. The Shimane CoHRE study conducted by Shimane University in Japan worked in collaboration with Okinoshima town in the Shimane prefecture to identify potential factors of healthy aging among rural residents. Japanese people with national health insurance are eligible for health checkups once a year in their municipality of residence. The health checkup including face-to-face structured interviews was conducted by trained nurses or public health nurses, as recommended by the Japanese Ministry of Health, Labor, and Welfare. Furthermore, additional health data, i.e., measurements for sarcopenia, were collected from the same participants in the health checkup. The inclusion criteria were living in Okinoshima town, participating in the health checkup, and agreeing to participate in the Shimane CoHRE Study. Overall, 789 older adults participated in health checkup in 2018. After excluding participants who did not agree to participate in the Shimane CoHRE study (*n* = 12) and participants with missing data (driving status, *n* = 1; exercise habits, *n* = 16; and sarcopenia, *n* = 22), we analyzed data from 738 participants. The study protocol was approved by the Ethics Committee at Shimane University (#2888), and written informed consent was obtained from all participants before enrollment.

### 2.2. Sarcopenia

Sarcopenia has been defined as a clinical disorder in the WHO’s International Classification of Diseases (ICD) Version 10 (ICD-10-CM (M62.84)) in 2016 and is based on objectively measured muscle mass and function [9]. The Asian Working Group for Sarcopenia 2019 consensus defined sarcopenia as “age-related loss of muscle mass, plus low muscle strength, and/or low physical performance” and specified cutoffs for each diagnostic component. Thus, we used skeletal muscle mass index (SMI), grip strength, and walking speed to identify possible sarcopenia in this study.

Limb and trunk muscle mass were measured with a body composition analyzer using the bioimpedance method (MC-780A) (Tanita Corporation, Tokyo, Japan). SMI was calculated on the basis of the limb and trunk skeletal muscle mass divided by the square of the body height. SMI was applied on the basis of sex-specific cutoffs (<7.0 kg/m^2^ for men and <5.7 kg/m^2^ for women) [10]. Grip strength was measured using a Smedley-type handheld dynamometer (GRIPD) (Takei, Niigata, Japan). Maximum grip strength was applied based on sex-specific cutoffs (<28 kg for men and <18 kg for women) [10]. Comfortable walking speed was measured using a sheet-type pressure sensor (45.5 × 183 cm plate sensor; Healthwalk) (Kao Corporation, Tokyo, Japan) placed in the center of a 5-m walkway [11]. Participants were asked to walk freely along a 7-m course and return to the starting point. On the course, acceleration and deceleration zones were set as 1-m at both ends. The average walking speed (m/s) of one round trip (i.e., two times) was used for the analysis. Slow gait speed was determined on the basis of a cutoff speed of < 1.0 m/s [10]. Sarcopenia was classified when there was low muscle mass together with either low muscle strength, low physical performance, or both, according to the 2019 Asian sarcopenia criteria [10].

### 2.3. Car Driving Status

Car driving status, assessed by a questionnaire, was based on the following question: “Do you have a valid driving license and usually drive a car?” (yes = driver, no = non-driver) [12].

### 2.4. Living Arrangement

Living arrangement was assessed by a questionnaire and classified as single household or non-single household. Single household was defined as living alone based on marital status [13].

### 2.5. Covariates

We included the following variables as covariates: sex (men or women), age (continuous), current smoking habits, current alcohol consumption habits, exercise habits, and a number of current chronic diseases collected from a list of 13 items, i.e., hypertension, hyperlipemia, diabetes, hyperuricemia, stroke, heart disease, vascular disease, kidney disease, hepatic disease, gastrointestinal disease, endocrine disease, osseous disease, and cancer. Current smoking habits were assessed by the following question: “Are you a current regular smoker?” (yes or no). Current alcohol consumption habits were assessed by the following question: “How often do you drink? (sake, shochu, beer, liquor, etc.)” (if every day or sometimes = yes, if rarely drink or cannot drink = no). Exercise habits were assessed by the following question: “Do you engage in regular physical exercise or sports to improve your health (including agricultural activities)?” (yes or no).

### 2.6. Statistical Analysis

Health checkup data, including face-to-face interviews and the measurements for sarcopenia, were integrated into a whole using anonymous identification numbers for analysis.

Descriptive statistics showed the characteristics of study participants. Between-group differences were examined using the χ^2^ test for categorical variables and analysis of variance test for continuous data. To examine the association of car driving status and living arrangement with sarcopenia in older adults, logistic regression analyses were performed to estimate the odds ratios (ORs) and 95% confidence intervals (CIs). Four groups were set to determine combined conditions of car driving status and living arrangement (driver and living with others; driver and living alone; non-driver and living with others; non-driver and living alone), with the driver and living with others group as reference. Analyses were conducted in crude (Model 1 and Model 2) and adjusted (Model 3, Model 4, and Model 5) models. Statistical analyses were performed using IBM SPSS Statistics for Windows, Version 23 (IBM Corp., Armonk, NY, USA). For all analyses, *p*-values less than 0.05 were considered statistically significant.

## 3. Results

Table 1 shows the participants’ characteristics. Among the 738 participants, 11.2% were classified as sarcopenia. Sex, age, current smoking habits, current alcohol drinking habits, number of current chronic diseases, grip strength, walking speed, and sarcopenia were significantly different among the four groups (driver and living with others; driver and living alone; non-driver and living with others; non-driver and living alone). 

Table 2 shows the results of the logistic regression analyses. In the null model (Model 1), non-drivers were more likely to be diagnosed with sarcopenia than those who were drivers (OR = 2.89; 95% CI, 1.80–4.62). In the null model (Model 2), there were no significant associations between living arrangement and sarcopenia. In the adjusted model (Model 3), a similar association was observed as in Model 1 (OR = 1.69; 95% CI, 0.92–3.09), but this result was not statistically significant. The adjusted model (Model 4), including living arrangement as the explanatory variable, showed no significant associations of living arrangement with sarcopenia, while among four groups in the adjusted model (Model 5), participants who were in the non-driver and living alone group had a significantly higher OR of sarcopenia (OR = 2.21; 95% CI, 1.02–4.80) than the reference group (driver and living with others). Age was also statistically associated with sarcopenia (OR = 1.13; 95% CI, 1.09–1.18), but sex, current smoking habits, current alcohol drinking habits, exercise habits, and current chronic diseases were not significantly associated with sarcopenia.

## 4. Discussion

This study is the first to examine the combined conditions of car driving status and living arrangement with sarcopenia among community-dwelling older adults in Japanese rural areas. Although no significant effect of car driving status on sarcopenia was found independently (Model 3), combined conditions showed that the non-driver and living alone group had a significantly higher OR of sarcopenia than the reference category (driver and living with others). These findings suggest that both factors (i.e., car driving status and living arrangement) should be assessed in order to prevent sarcopenia in Japanese rural areas. 

A previous study has reported that driving limitations are associated with decreased out-of-home activity levels [4]. Furthermore, former drivers are more likely to experience increased social isolation and isolation is not always counteracted by access to alternative public transportation [14]. Thus, it is possible that driving limitations may lead to psychological consequences that may lead to declining social activities. Although further intervention studies are required, it is essential to avoid isolation by providing social support and social relationships with others, as well as to develop alternative transportation strategies for the prevention of sarcopenia in the rural settings of Japan.

During the last decade, there have been growing interests in the effect of neighborhood environments on health outcomes [15]. For example, a longitudinal study found that availability of both health-damaging resource, i.e., availability to the fast-food restaurants was predictor of both coronary heart disease and stroke [16]. Comparatively little research has examined the association between neighborhood environments and sarcopenia [17,18,19]. It is possible that the risk of sarcopenia could be decreased beyond the inconvenience of transportation if older adults who live alone are able to access health-promoting resources, e.g., food shops [20]. Therefore, further analyses that include environmental factors may be worthwhile.

This study has some limitations. First, we used a cross-sectional study design, which precludes the possibility of causal inference. For example, it is possible that older adults with sarcopenia stop driving a car. Second, selection bias may have occurred, although the participants were recruited from multiple centers in the study town. Third, the number of current chronic diseases in this study derived from self-reported data, which may have led to underestimations of health problems. Finally, our results could be influenced by other unmeasured risk factors, such as depression and socio-economic status.

We believe that despite these limitations, the findings of this study are a useful basis for future discussions in other countries that will face the concerns of rapid aging. Given the increasing older adult population, driving safety is especially relevant [1]. For example, the number of driver’s license holders aged 75 or over in Japan nearly doubled from 3,240,000 in 2009 to 5,830,000 in 2019, and elderly drivers aged 75 or over often have accidents with the vehicle alone (e.g., single-car collisions or leaving the road) [21]. One option to reduce accidents by elderly drivers is to encourage them to surrender their driving licenses. Our findings, however, suggest that we need to take a broader view of the impact of driving cessation to promote the independence of the elderly. 

## 5. Conclusions

This study found that being a non-driver and living alone is associated with a greater risk of sarcopenia among older adults living in a rural area of Japan. Our findings suggest that the consideration of both driving status and living arrangement are important in the formulation of public health strategies to prevent sarcopenia in rural Japanese areas.

## Figures and Tables

**Table 1:** Participants’ characteristics.

	Total	Drivers		Non-Drivers		
		Living with Others	Living Alone	Living with Others	Living Alone	*p*-Value
	738	392 (53.1)	62 (8.4)	210 (28.5)	74 (10.0)	
Sex						**<0.01**
Men	273 (36.8)	209 (53.3)	23 (37.1)	31 (14.8)	10 (13.5)	
Women	465 (63.2)	183 (46.7)	39 (62.9)	179 (85.2)	64 (86.5)	
Age	73.7 (8.1)	71.0 (8.1)	72.6 (5.8)	77.4 (6.7)	79.1 (6.7)	**<0.01**
Current Smoking						**<0.01**
No	695 (94.2)	362 (92.3)	54 (87.1)	205 (97.6)	74 (100.0)	
Yes	43 (5.8)	30 (7.7)	8 (12.9)	5 (2.4)	0	
Current Alcohol Drinking						**<0.01**
No	490 (66.4)	215 (54.8)	33 (53.2)	178 (84.8)	64 (86.5)	
Yes	248 (33.6)	177 (45.2)	29 (46.8)	32 (15.2)	10 (13.5)	
Exercise						0.56
No	214 (29.0)	120 (30.6)	20 (32.3)	54 (25.7)	20 (27.0)	
Yes	524 (71.0)	272 (69.4)	42 (67.7)	156 (74.3)	54 (73.0)	
Number of Current Chronic Diseases	1.7 (1.4)	1.5 (1.4)	1.9 (1.2)	2.0 (1.5)	1.9 (1.3)	**<0.01**
Skeletal Muscle Mass Index (Men: <7.0 kg/m^2^, Women: <5.7 kg/m^2^)	180 (24.4)	84 (21.4)	13 (21.0)	58 (27.6)	25 (33.8)	0.07
Grip Strength (Men: <28 kg, Women: <18 kg)	100 (13.6)	32 (8.2)	10 (16.1)	30 (14.3)	28 (37.8)	**<0.01**
Walking Speed ( <1.0 m/s)	183 (24.8)	66 (16.8)	11 (17.7)	77 (36.7)	29 (39.2)	**<0.01**
Sarcopenia						**<0.01**
No	655 (88.8)	362 (92.3)	60 (96.8)	178 (84.8)	55 (74.3)	
Yes	83 (11.2)	30 (7.7)	2 (3.2)	32 (15.2)	19 (25.7)	

^1^ Statistical significance of the differences between groups was determined using the χ^2^-test for categorical data and the analysis of variance test for continuous data.^2^ Values in boldface show significance (*p* < 0.05).

**Table 2:** Associations of car driving status and living arrangement with sarcopenia among community-dwelling older adults.

	Model 1	Model 2	Model 3	Model 4	Model 5
	Crude model	Crude model	Adjusted model	Adjusted model	Adjusted model
	OR (95% CI)	OR (95% CI)	OR (95% CI)	OR (95% CI)	OR (95% CI)
Car driving status					
Driver	1 (reference)		1 (reference)		
Non-driver	**2.89 (1.80, 4.62)**		1.69 (0.92, 3.09)		
Living arrangement					
Living with others		1 (reference)		1 (reference)	
Living alone		1.59 (0.93, 2.71)		1.28 (0.72, 2.28)	
Driving status and living arrangement					
Driver and living with others					1 (reference)
Driver and living alone					0.41 (0.09, 1.83)
Non-driver and living with others					1.30 (0.67, 2.52)
Non-driver and living alone					**2.21 (1.02, 4.80)**
Sex					
Men			1 (reference)	1 (reference)	1 (reference)
Women			0.63 (0.33, 1.18)	0.75 (0.42, 1.33)	0.65 (0.35, 1.24)
Age			**1.14 (1.09, 1.18)**	**1.15 (1.10, 1.19)**	**1.13 (1.09, 1.18)**
Current smoking					
No			1 (reference)	1 (reference)	1 (reference)
Yes			0.83 (0.18, 3.84)	0.79 (0.17, 3.72)	0.90 (0.19, 4.23)
Current alcohol drinking					
No			1 (reference)	1 (reference)	1 (reference)
Yes			0.89 (0.47, 1.69)	0.82 (0.44, 1.55)	0.90 (0.47, 1.71)
Exercise					
No			1 (reference)	1 (reference)	1 (reference)
Yes			1.01 (0.58, 1.76)	1.01 (0.58, 1.76)	1.00 (0.57, 1.75)
Current chronic diseases			1.04 (0.88, 1.23)	1.05 (0.88, 1.24)	1.05 (0.89, 1.25)

Values in boldface show significance (*p* < 0.05).
